# Complete Genome Sequence of Community-Acquired Methicillin-Resistant Staphylococcus aureus Strain RJ1267, Isolated in Shanghai, China

**DOI:** 10.1128/MRA.00244-20

**Published:** 2020-04-30

**Authors:** Xianchun Zong, Dehua Liu, Min Li, Baolin Sun

**Affiliations:** a Mudanjiang Normal University, Mudanjiang, Heilongjiang, China; b Department of Oncology, The First Affiliated Hospital, University of Science and Technology of China, Hefei, Anhui, China; c Department of Laboratory Medicine, Renji Hospital, School of Medicine, Shanghai Jiaotong University, Shanghai, China; University of Rochester School of Medicine and Dentistry

## Abstract

Staphylococcal pathogens, especially multidrug-resistant Staphylococcus aureus, are responsible for various clinical infections. Multilocus sequence type 630 (ST630) methicillin-resistant Staphylococcus aureus has been shown to have augmented pathogenicity in humans. In this announcement, we report the complete genome sequence of community-acquired methicillin-resistant strain RJ1267 of Staphylococcus aureus ST630.

## ANNOUNCEMENT

Staphylococcus aureus is a major human pathogen that can cause a variety of clinical infections worldwide, ranging from minor localized infections to more severe invasive illnesses (1–10). The use of different types of antibiotics over the years has led to the emergence of methicillin-resistant S. aureus (MRSA) strains, which are often resistant to other classes of antibiotics (11).

Strain RJ1267 of Staphylococcus aureus multilocus sequence type 630 (ST630) is a human clinical isolate from Renji Hospital, affiliated with Shanghai Jiaotong University (Shanghai, China). RJ1267 is an MRSA strain that is resistant to multiple antibiotics, including methicillin, penicillin, oxacillin, and clindamycin (12). The RJ1267 clone was initially isolated among methicillin-susceptible S. aureus strains, as described for the S. aureus multilocus sequence typing (MLST) database (https://pubmlst.org/saureus/), and has not been reported in association with human infection. ST630 is a close relative of ST8, a type described as the putative ancestral genotype of another subgroup within clonal complex 8 (CC8) (13). We used this strain as a model system for comprehensive identification of genes essential for bacterial drug resistance (14). Here, we present the complete genome of MRSA strain RJ1267 for performing further comparative genomic studies and determining the impact of genetic background on the function of drug resistance genes. S. aureus strain RJ1267 was isolated as a colony from a sheep blood agar plate and inoculated into tryptic soy broth at 37°C with shaking (220 rpm). Genomic DNA from the cultured isolate was extracted using the Wizard genomic DNA purification kit (Promega, San Luis Obispo, CA, USA) according to the manufacturer’s instructions. The complete genome of S. aureus strain RJ1267 was sequenced using a PacBio Sequel I platform and a BGISEQ-500 platform at the Beijing Genomics Institute (BGI) (Shenzhen, China). A sheared large-insert library was generated using Covaris g-TUBES and the SMRTbell template preparation kit v1.0 and then was sequenced with PacBio RS II sequencing technology using one single-molecule real-time (SMRT) cell.

A total of 8,546,548 raw paired-end reads with a length of 150 bp were obtained from the BGISEQ system, and low-quality reads (>40% bases with a quality score lower than 20 and N content of >10%) were filtered using SOAPnuke v2.0 (15). In total, 300,722 subreads were obtained from the PacBio system, and the subreads with less than 1 kb were removed. The *N*_50_ and *N*_90_ values for the subreads were 8,698 bp and 6,364 bp, respectively. Hybrid genome assembly using the trimmed BGISEQ reads and filtered PacBio subreads was performed using the Unicycler v0.4.8 pipeline (SPAdes v3.13.2, minimap, Racon v1.4.10, and Pilon v1.23) (16–20) with default parameters. The error-corrected assembly was tested for possible circularity using Circlator v1.5.5 (21); all steps were executed with the following parameters: merge_min_id, 85; merge_breaklen, 1,000; assembler, SPAdes. After assembly, the GC content was determined with QUAST v4.4 (22), and gene annotation was accomplished using Prokka v1.14.0 (23) (with the parameters prefix metagG, metagenome, kingdom *Bacteria*, and genus *Staphylococcus*).

Two circular contigs resulted from hybrid assembly of the ST630 reads, i.e., the chromosome and a plasmid (2,473 bp). The resulting chromosome was 2,899,307 bp long, with 2,878 identified genes, of which 2,796 were coding sequences and 82 were RNA genes (19 rRNAs, 62 tRNAs, and 1 transfer mRNA). The GC content was 32.75%.

### Data availability.

The genome sequence and associated data for RJ1267 were deposited under GenBank accession numbers CP047321 and CP047322, BioProject accession number PRJNA598122, BioSample accession number SAMN13698767, and SRA accession numbers SRR10807891 (BGISEQ) and SRR10807892 (PacBio).
